# Cryo electron microscopy to determine the structure of macromolecular complexes

**DOI:** 10.1016/j.ymeth.2015.11.023

**Published:** 2016-02-15

**Authors:** Marta Carroni, Helen R. Saibil

**Affiliations:** ISMB, Birkbeck College, Malet St, London WC1E 7HX, UK

## Abstract

Cryo-electron microscopy (cryo-EM) is a structural molecular and cellular biology technique that has experienced major advances in recent years. Technological developments in image recording as well as in processing software make it possible to obtain three-dimensional reconstructions of macromolecular assemblies at near-atomic resolution that were formerly obtained only by X-ray crystallography or NMR spectroscopy. In parallel, cryo-electron tomography has also benefitted from these technological advances, so that visualization of irregular complexes, organelles or whole cells with their molecular machines *in situ* has reached subnanometre resolution. Cryo-EM can therefore address a broad range of biological questions. The aim of this review is to provide a brief overview of the principles and current state of the cryo-EM field.

## Highlights

• Structural biology.
• Cryo electron microscopy.
• Macromolecular complexes.
• Single particle analysis.

## 1 Introduction: biological structure determination by electron microscopy

The development of three-dimensional (3D) reconstruction from electron microscopy (EM) images was based on methods in X-ray crystallography, and the earliest 3D structures were obtained from ordered specimens such as 2D crystals, helical arrays and icosahedral viruses [1], [2], [3], [4]. A parallel development, which came to fruition much later, was in 3D reconstruction of arbitrary objects by electron tomography and of isolated assemblies of any symmetry by single particle analysis [5], [6], [7].

A major limitation of these methods was formerly the need for sample staining and dehydration, in order to provide contrast and withstand the vacuum inside the column of the electron microscope. This was overcome with the development by Dubochet and colleagues, based on earlier work by Taylor and Glaeser, of a simple method for rapid freezing to produce vitrified samples, in which a thin layer of aqueous solution is solidified by cooling too rapidly to allow ice crystallization [8], [9], [10]. The vitrified samples are stable in the column vacuum and the low temperature also slows the rate of radiation damage, the other main limitation in EM of biological complexes.

With the development of microscope technology (stable, low temperature sample holders, more coherent electron sources, low dose imaging) and of image processing methods, the structure of a membrane protein showing backbone and some side chain information was obtained from well-ordered 2D crystals [2]. The field advanced slowly but steadily, broadening the range of ordered samples and yielding backbone, and eventually, side chain resolution for rigid icosahedral viruses [11], [12], [13]. Single particle analysis and tomography were also developing in parallel, benefiting from the microscope improvements and generating new approaches for 3D reconstruction. The recent, dramatic advances leading to an increasing number of near-atomic structures (<4 Å resolution) of single particles have brought the field into much greater prominence.

## 2 Sample preparation and imaging conditions

To understand how biological systems work from the biochemical to the cellular scale it is necessary to determine how molecules or organelles assemble in 3D into their functional forms. EM allows the direct visualization and 3D reconstruction of individual purified molecules or complexes of molecular mass greater than ∼100 kDa, isolated assemblies, tissue sections, organelles or even whole cells, provided that they are thin enough (<1 μM) to transmit the electron beam. The structural information in the recorded image arises from elastic interactions between the electrons and the specimen. It can be defined as the projected Coulomb potential of the sample or simply as the 2D projection, along the beam direction, of the specimen density. The intensity of electron scattering is small for biological molecules. Biological specimens thus yield low contrast images, and are also extremely sensitive to damage by the electron beam [14], [15], [16].

Due to the strong interaction of electrons with matter, the microscope column is kept under high vacuum. Therefore the sample must be imaged in a solid state. Isolated assemblies can be prepared by staining with a heavy metal salt solution followed by drying [17]. This method is called negative staining, since the stain forms a negative image around the surface of the structure [18], [19]. It has been used for over 4 decades and it is still common for preliminary sample visualization, especially of small particles (<200 kDa), since it is quick and gives high contrast images. To obtain *ab initio* 3D reconstructions of macromolecules of unknown structure it is usually a good idea to check the sample by negative stain. However, staining and drying can introduce artifacts such as flattening or partial staining, and limit the resolution to ∼20 Å due to the stain granularity and lack of penetration inside the structure [20].

In order to observe the full structure in the hydrated state, cryo-EM is essential. For cryo-EM, a small aliquot of sample in solution or suspension is applied to an electron microscopy grid and is blotted to a thin layer and immediately plunged into liquid ethane (around −180 °C), so that the molecules get trapped in a layer of vitrified water [10]. Ideally the ice should be just thick enough to accommodate the particles without distortion. This approach can also be applied to very thin regions of adherent cells. However, thicker cells and tissue samples cannot be vitrified by plunge freezing and they are also too thick to be imaged intact. Traditionally, cells and tissues are prepared by chemical fixation, which involves crosslinking, dehydration, and staining with heavy metal solutions, followed by plastic embedding and sectioning. Most of our information on cell structure has been obtained with this methodology. However, cells and tissues can be imaged in their frozen state if they are prepared by high pressure freezing, in which 100–200 μM thick cell pellets or tissue slices are vitrified by liquid nitrogen cooling in a highly pressurized chamber [21], [22]. Cryo-sectioning or focused ion beam milling is then required to produce thin specimens from which 3D structures can be determined by electron tomography [23], [24], [25].

Because of their high sensitivity to electron damage, biological cryo-EM samples must be imaged under low dose conditions, with an electron dose of 10–20 e^−^/Å^2^ to preserve high-resolution details [26]. The resulting images have very a low signal to noise ratio, compounding the problem that unstained cryo samples have very little intrinsic contrast. Biological macromolecules can be considered as weak phase objects, because their very weak scattering is mainly manifested by small phase changes in the scattered beam [[14], chapter 2]. These small phase changes can be turned into visible contrast by defocusing the objective lens. Electron lenses have spherical aberration, and defocussing introduces differences between the scattered and unscattered rays that result in visible contrast. However, this also causes other changes to the image, including the loss of information at some spatial frequencies and phase reversals in alternating bands. These changes are described by the contrast transfer function (CTF), an oscillating function with regions of zero transmission, resulting in loss of certain frequency bands from the image. In addition, the CTF amplitude declines at higher frequencies, causing progressive loss of the higher resolution information. These losses depend on the defocus used for each image, as well as general optical parameters such as coherence of the electron source. Therefore, cryo-EM imaging involves a trade-off between contrast and resolution. In practice, the distortions introduced by the CTF can be corrected, but images must be recorded at a range of defocus settings in order to fill in the missing information when they are combined into the reconstruction.

Current transmission electron microscopes are very stable, capable of imaging to a resolution better than 2 Å with bright and coherent beams generated by field emission guns and electronic control for automated data collection. However, for vitrified biological samples, the resolution is ultimately limited by electron beam damage. Collection of high quality data on such samples remains challenging and expertise in microscope alignment, and in selection of dose, defocus and other imaging settings are important [27], [28].

## 3 Recent advances in cryo-EM imaging

The recent, spectacular advance in resolution of single particle cryo EM is mainly due to the development of direct electron detectors [29], [30], [31], [32]. The effect on the EM field is similar to the effect on macromolecular crystallography of the replacement of photographic film with electronic detectors for X-rays.

Previously, high resolution EM imaging relied either on photographic film, which is extremely slow, cumbersome, and limited in sensitivity, or else more convenient but indirect detection by charge-coupled devices (CCDs). CCDs are inferior in resolution to film but allow automated data collection [33], [34]. The main problem is that the incident electrons must be converted to visible light for electronic detection by the CCD sensor, and this conversion entails serious losses of resolution and sensitivity. In contrast, the current generation of direct detectors record the incident electrons in a thin, sensitive layer so that the signal is not scattered into surrounding pixels. They have much improved sensitivity, measured as detective quantum efficiency (DQE), which is defined as SNR^2^(k)_out_/SNR^2^(k)_in_, where SNR is the signal to noise ratio and k is the spatial frequency. Moreover, the readout is much faster, enabling images to be acquired as a series of movie frames at 17–400 frames per second.

**Fig. 1:**
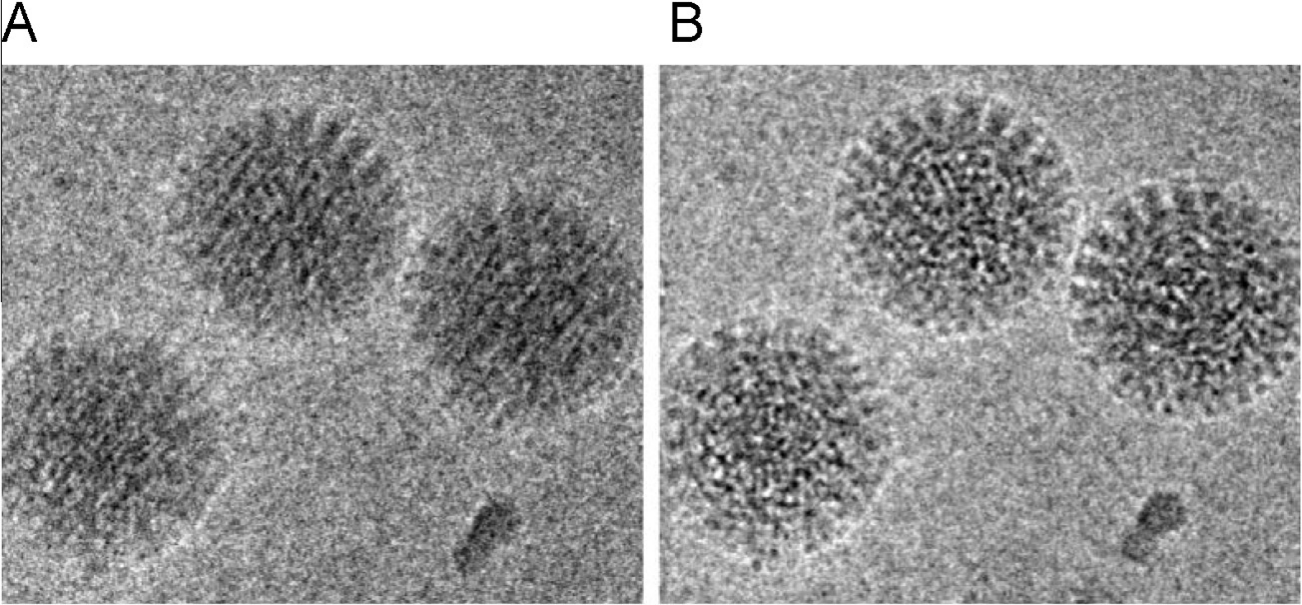
Motion correction and recovery of high-resolution information Average of frames of rotavirus particles before (A) and after (B) translational alignment. Features are blurred before alignment. Image from [38], Elsevier copyright, Inc.

This movie mode provides two major advantages. First, and most importantly, specimen movement, beam-induced or from other causes, can be corrected, thus allowing the recovery of high-resolution details that were poorly transmitted in single images (Fig. 1) [35], [36], [37], [38], [39]. Specimen movement during data collection was previously a major limitation to the resolution and efficiency of cryo EM studies. The fast readout and high DQE of direct detectors allows the correlation of features between movie frames, each of which has an extremely low electron dose (1 e^−^/pixel), so that specimen displacements can be tracked and corrected [40].

A second consequence of high-speed detection is that it enables electron counting within a reasonable total exposure time. Using the same principle as super-resolution optical microscopy, individual frames are recorded with very sparse electron events, so that electrons can be individually detected, scaled to unit intensity and their peak positions located to sub-pixel accuracy [41], [42], [43]. Moreover, the ability to count single electron events allows the rejection of the Landau noise coming from the primary electron deposition [40]. Drift correction greatly improves the DQE at high resolution, whereas electron counting causes a big improvement to the low-resolution signal [42], [43], [44]. The drift correction improves the resolution obtainable, and the stronger low-resolution signal has a big impact on detection and alignment of smaller objects (<∼200 kDa), which would otherwise be intractable by cryo-EM. An experimental approach to reducing specimen movement, particularly in the vertical direction, is the use of gold grids with a gold support foil, instead of copper grids coated with a layer of carbon [45].

Now that high-resolution 3D reconstructions of purified macromolecular complexes can be obtained, we can envisage the recovery of high-resolution information from structures inside the cell by cryo electron tomography [23], [46]. Using energy filters, inelastically scattered electrons can be excluded from the image, thus improving the signal to noise ratio [47]. Inelastic scattering is significant in thick and highly tilted specimens such as the ones used in electron tomography. The improvement in low resolution DQE from electron counting is also very helpful for cryo tomography, since it makes features clearer in the tomograms without any averaging.

Another innovation, which greatly improves image contrast is the use of Zernike phase contrast as in light microscopy [48]. A 90° phase shift is introduced between the scattered and unscattered electrons to convert phase differences into amplitude differences, removing the need to defocus the objective lens. Promising results have been obtained in electron tomography of cellular specimens [46]. Several types of phase plates have been developed for EM; of these, the Volta phase plate seems best for ease of use [49].

Spherical aberration correction, which is used in high-resolution materials science imaging [50], has been used for a high-resolution reconstruction of ribosome structure [51]. The importance of this correction remains to be demonstrated for biological EM.

## 4 Structure determination by single particle analysis

In parallel with the advances in instrumentation, software developments have also contributed to the improvements in resolution. Although the basic tool for detecting similarity is cross-correlation, new approaches use Bayesian statistical methods taking account of noise, and address the problem of sample heterogeneity in a more automated way than earlier methods. The programs Relion [52] and Frealign [53], [54], using these methods, have generated many of the recent high-resolution structures [55]. The advances in single particle analysis have fuelled the exponential growth in number of structures deposited in the EM databank [56]. Automated data collection has also facilitated the task of collecting sufficiently large data sets for reliable statistical analysis. Freely available automatic data acquisition systems include Serial EM [57], Leginon [58], and UCSFImage4 [59]. An example of a commercial system is EPU (FEI, Eindhoven).

**Fig. 2:**
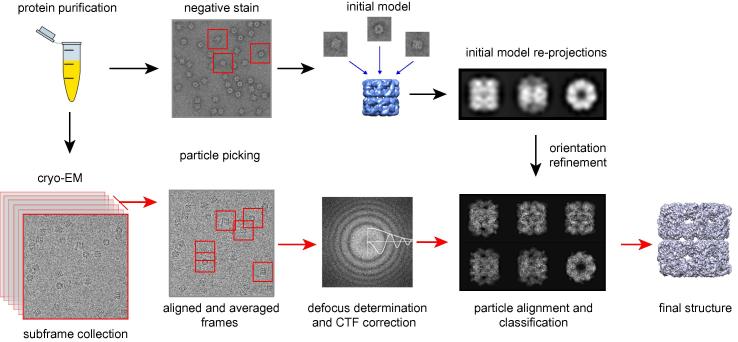
Schematic of single-particle reconstruction *Protein purification.* High purity of the sample is important, as in crystallography. *Negative stain* is useful to clearly visualize the sample and check its homogeneity, especially for small particles. Particles are boxed from the micrographs, centered and aligned. Classification and averaging give improved SNR, and class averages can be used to obtain a low-resolution *initial model* by common lines or tilt methods. In *cryo-EM*, the vitrified sample is imaged by collecting movie frames that are aligned for motion correction and then averaged. *Defocus determination and CTF correction* are done on motion-corrected averaged images. After alignment, classification and cleaning of the dataset, particles are assigned orientations by projection matching to the initial model. *Orientation refinement* is performed iteratively until the structure converges.

A general overview of the single particle analysis procedure is shown in Fig. 2 and explained below. More detailed information can be found in Orlova & Saibil, 2011 [60] and in more recent reviews by Cheng et al., 2015 [61] and Carazo et al., 2015 [62]. Ideally, a single particle sample will contain a good distribution of separate (i.e., not aggregated), identical particles in random orientations in the vitreous ice. It may of course take years of biochemistry and trial and error experimentation to reach this point. Given such a sample, a data set of ∼1000 image movies taken on an FEG microscope at a variety of defocus values, each with a few hundred (asymmetric) particles, should contain all the information needed for a high resolution 3D reconstruction. In practice, there is often heterogeneity in the particle population, and this greatly increases the amount of data needed to reach a given resolution. The first step in processing is movie alignment to produce an averaged drift-corrected image from each movie. Conversely, symmetrical structures require correspondingly fewer images. The initial frames have the least radiation damage and contain high-frequency information, but usually suffer the most beam-induced movement. The final frames have greater loss of high spatial frequencies due to the accumulated electron dose, but they can still provide useful contrast at low frequencies. A simple or weighted average of the aligned frames can be used, with the elimination of 2–3 initial and final frames, retaining those with least beam-induced movement, up to a dose of 20–25 e/Å^2^ [39], [63]. Defocus determination is done on the motion-corrected average image, by fitting a CTF model to the observed oscillations in the image amplitudes [64], [65]. With the determined defocus, CTF correction can be done to restore the correct phases at all spatial frequencies with detectable signal. The amplitudes can be restored later in the processing, at the stage of merging images taken at different defocus. Then the particles are picked, usually by an automated procedure, to produce the data set for particle alignment and reconstruction. In general, automated particle picking generates data sets containing a significant number of “junk” particles, and these must be purged from the data set during the initial stages of processing. The particles are extracted in boxes of diameter at least twice the maximum dimension of the object, to give adequate sampling of the transform during the alignment search. Particle picking (both manual and automatic) and boxing can be done with most commonly used software for single particle analysis (e.g. EMAN-2, Xmipp, Relion).

Automatic picking procedures generally give particles fairly centered in their boxes. Otherwise, particles must be centered prior to alignment and classification. One approach for initiating reference-free alignment is to align all particles to their rotationally averaged sum. With a few iterations, this approach (or use of random subsets of the data as initial references) gives reasonably good centering, but it is more difficult with elongated or irregularly shaped objects. In order to get a first understanding of the data set, it is useful to sort the images into similar subsets, or classes, to generate averages with higher SNR than the original images. This is done by statistical comparison of all images, pixel by pixel, typically using principal component or multivariate statistical analysis to find the principal variations within the dataset [66], [67]. These principal components are then used to classify the images according to their features. The resulting class averages can serve as reference images in a multi-reference alignment, in which all images are cross correlated to all references to find the best match for each image and to refine the classification. These steps are iterated to improve the sorting and alignment. At this stage it should become clearer if the sample is homogeneous and has well distributed orientations. Particles can also be aligned and classified at the same time, using maximum likelihood algorithms that assign a specific particle to a set of classes with a probability distribution. Alignment and classification can be performed with a variety of software packages with different degrees of manual supervision. There are efforts to automate data processing workflows using pipelines of procedures from different software packages (Scipion, http://scipion.cnb.csic.es; Appion [68]).

Provided that there is a good distribution of view orientations, an initial 3D reconstruction of the molecule can be obtained. *Ab initio* structure determination is challenging and it can help to obtain an initial model from negative stain data in which the particles are clearly visible. Similarly, is it useful to have class averages with improved SNR for initial 3D reconstruction. To overcome the problem of angle assignment when it comes to building a completely *ab initio* structure, one option is to generate an initial model by electron tomography in which a series of views of the same area are collected over the whole range of tilts (e.g. −60° to 60°) [69]. Because of the accumulated electron dose and the missing data due to limited tilt, the resolution is limited, making this approach less feasible for smaller structures. Similar methods, which can be thought as hybrid between tomography and single-particle approaches, are random conical tilt (RCT) [70] and orthogonal tilt reconstruction (OTR) [71]. In RCT, pairs of images are collected, one after tilting the stage at some angle between 45° and 60° and the other untilted. Since the tilt angle is experimentally set and the in-plane rotation angle can be found by alignment, a fairly reliable model can be obtained. For particles with a preferred orientation, the limitation in tilt angle results in a missing cone of data. If the particles have a distribution of orientations, multiple 3D structures obtained by RCT can be averaged to generate an isotropically resolved starting model. OTR relies on having a good distribution of particle orientations to fill in the missing data, and uses −45°, 45° tilt pairs.

*Ab initio* reconstructions can also be obtained by computationally determining the relative orientation of particles based on the common line theorem that states that every pair of 2D projections of the same 3D structure has at least one 1D (line) projection in common. In Fourier space, the Fourier transforms of the 2D projections are planes that pass through the origin of the 3D Fourier transform of the object, and their intersection is the common line. With three 2D projections it is therefore possible to establish their relative orientations. However, 3D reconstruction by common lines is unreliable with individual, noisy images. Classification and averaging are needed and the presence of symmetry greatly facilitates the procedure. The first implementation of common lines was done on icosahedral viruses [4], [72]. Common line assignment of orientations is implemented in various software packages and can be done both in Fourier (EMAN, SIMPLE) and real space (IMAGIC), generally using class averages rather than single images [67], [73], [74].

If the structure is related to a known 3D structure, the orientation parameters can be determined by projection matching, in which reprojections of the model structure are used as references for alignment of the data set by cross-correlation [66], [75], [76] Each particle is assigned the orientation of the best matching reference. Once orientations are assigned and a first 3D map is reconstructed, it can be refined by further cycles of common lines analysis or projection matching with finer angle search.

A more recent approach to refinement is based on maximum likelihood algorithms [77], [78], [79] and Bayesian analysis [52], [80] in which particles are not assigned to a single class, but are given instead a probability distribution of membership in a set of 3D classes. The maximum likelihood approach in Relion includes a model for the noise, and automates judgments about filtering and weighting that were formerly done by expert users [81]. The Bayesian approach, combined with the use of direct detectors, has been extremely successful in generating the unprecedented stream of high-resolution single particle structures. However, for challenging structures, these programs still require a reliable starting model. The maximum likelihood approach has recently been implemented in the *ab initio* structure determination program PRIME [82].

Subtomogram averaging is a 3D version of single particle analysis in which subvolumes, rather than 2D projections, are extracted from tomograms for alignment and classification. Briefly, tilt series of the sample containing multiple copies of the macromolecular complex of interest, either in cell or purified, are taken and tomograms are reconstructed. Programs for automated collection of tomograms have been developed, such as the popular Serial EM [57] or TOM^2^ [83], FEI Tomo4 (FEI, Eindhover) and UCSF Tomo [58]. Because of the tilting, CTF correction is more complex than for untilted 2D projections, but it is implemented in various software packages [84], [85], [86], [87]. Subvolumes containing the structure of interest are extracted, aligned and classified. By averaging copies of the structure that were present in different orientations in the original tomogram, the missing wedge is filled in and the resolution is improved. As for single particle analysis, a large number of homogeneous subvolumes facilitates the analysis and is more likely to yield high-resolution molecular features [88].

## 5 Resolution measurement and map validation – not fully solved problems

The resolution of the refined 3D structures is estimated by calculating the Fourier shell correlation (FSC) between two volumes obtained by splitting the dataset into halves. The FSC is a measure of reproducibility as a function of spatial frequency. The resolution of the reconstruction is defined as the FSC value at a given threshold [89] and it is nowadays agreed that the FSC should be calculated by correlating two volumes that have been independently reconstructed [81]. It is difficult to assess the quality and real resolution of an EM reconstruction and it is important to make sure that there is no over-fitting of the data. Over-fitting comes from the refinement of the noise rather than the signal present in the dataset and it can happen even when the two half datasets used for the estimation of the resolution are processed independently. This is because the processing can never be truly independent and other parameters, such as masking, also have an effect on the FSC. The reliability of a reconstruction can be tested by tilt pair validation, in which some pairs of images at 0° and at a modest tilt, around 20°, are recorded and the particle orientations determined by projection matching to the map. If the map is correct, this procedure should correctly determine the difference in tilt between the pairs of particles [90]. Other approaches are to filter out high resolution features from the reference map when starting the refinement, and to randomize the phases of the half maps beyond a cut-off frequency and check that the FSC drops to zero at frequencies higher than the cut-off [91]. The resolution is often not uniform, and in the case of tomography, it is anisotropic, with lower resolution along the beam direction. Local variations in resolution can be mapped onto a structure with the program Resmap [92]. There are combined efforts within the EM community to find standards for proper validation of 3D reconstructions, with ongoing discussions of the appropriate criteria [93].

## 6 Recent examples of biological structures determined by single particle analysis and subtomogram averaging

### 6.1 Rotavirus VP6 at 2.6 Å resolution

**Fig. 3:**
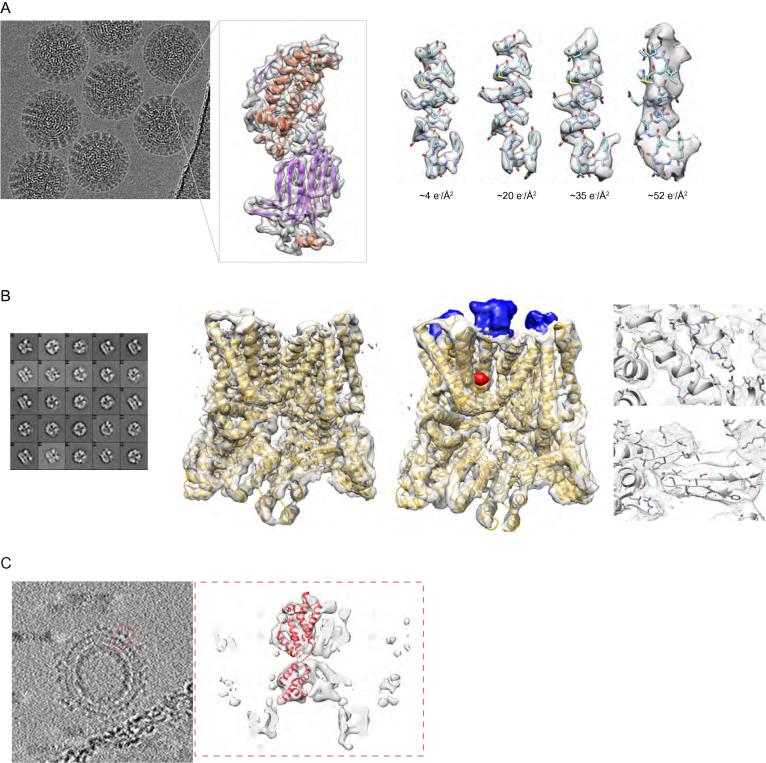
Examples of cryo-EM macromolecular reconstructions (A) An average of aligned frames of the rotavirus double-layered particle and 3D reconstruction of an extracted subunit. On the right are shown the effects of electron damage on a segment of the polypeptide chain (reproduced in part from [94] (EMD-6272)). (B) Structure of the TrpV ion channel with class averages on the left (reproduced from [98]; Nature Publishing Group, Inc.), structure without (EMD-5778) and with ligands (blue and red; EMD-5777) in the middle, details of fitted α-helices and β-strands on the right. (C) Subtomogram averaging of Gag protein from RSV retrovirus. On the left, averaged slices through the tomogram of immature viral particles (EMD-3102). In the right dashed box, subtomogram average of a Gag subunit with the fitted secondary structure (EMD-3101).

By optimizing the exposure rate and conditions of movie data collection Grant and Grigorieff [94] improved the resolution of their data on rotavirus double-layered particles (Fig. 3A). They performed radiation damage measurements similar to those carried out on 2D crystalline materials [95], [96], [97]. From this analysis, they determined an optimal exposure curve and used it to design a filter for radiation damage correction. This filter can be applied from the initial steps of processing, thus also helping in particle picking and frame alignment.

### 6.2 The TRPV1 ion channel

Sorting of heterogeneous conformations with the maximum likelihood method implemented in Relion [52] made it possible to obtain the structure of the 300 kDa TRPV1 ion channel at 3.4 Å resolution. This was the first high-resolution structure of a membrane protein obtained by single particle cryo-EM rather than crystallography [98]. TRPV1 is the receptor for the chili burning compound capsaicin, a general sensor for temperature changes in the cell, and is modulated by inflammatory agents. In another study the same group also obtained structures of the ion channel with two different ligands that clarified the ion gate opening mechanism [99]. The level of detail offered by these cryo-EM reconstructions is comparable to that in crystallographic structures. Moreover, the authors highlight the advantage of single particle cryo-EM over crystallography in flexibility of experimental conditions, allowing investigation of temperature effects on channel activity.

### 6.3 The Gag polyprotein: subtomogram average of purified macromolecular complexes

The Gag polyprotein is the major precursor protein of retroviruses including HIV. By subtomogram averaging on the retroviral model RSV, Schur et al. obtained a 7.7 Å resolution reconstruction of Gag, the highest so far obtained using this approach [100]. At this resolution helices are visible, and the map could be readily interpreted by flexible fitting with available crystal structures.

## 7 Conclusion

In conclusion, cryo EM is an increasingly important method in structural molecular and cell biology. The field is still developing and it is likely that there will be continuing advances in resolution, automation of data collection and ease of use. One difficulty is the very high cost of the top end equipment. This is being addressed by the development of centralized facilities that operate like synchrotron beam lines, for example at the Diamond light source in the UK [31]. Although centralized facilities alleviate the problems of high cost and infrastructure demands on individual institutions, they do not remove the need for in house equipment, to support the often lengthy development of cryo EM projects to a stage where high resolution data can be collected.
